# AM251 Suppresses Epithelial-Mesenchymal Transition of Renal Tubular Epithelial Cells

**DOI:** 10.1371/journal.pone.0167848

**Published:** 2016-12-09

**Authors:** Tomoyo Yoshinaga, Kenichiro Uwabe, Shoichi Naito, Kenichi Higashino, Toru Nakano, Yoshito Numata, Akio Kihara

**Affiliations:** 1 Laboratory of Biochemistry, Graduate School of Life Science, Hokkaido University, Sapporo, Japan; 2 Biomarker R&D Department, Shionogi & Co., Ltd., Toyonaka, Japan; 3 Global Innovation Office, Pharmaceutical Research Division, Shionogi & Co., Ltd., Toyonaka, Japan; 4 Corporate GxP Compliance Office, Shionogi & Co., Ltd., Toyonaka, Japan; 5 Drug Discovery Disease Laboratory, Shionogi & Co., Ltd., Sapporo, Japan; 6 Laboratory of Biochemistry, Faculty of Pharmaceutical Sciences, Hokkaido University, Sapporo, Japan; University of Colorado Boulder, UNITED STATES

## Abstract

Epithelial-mesenchymal transition (EMT) of renal tubular epithelial cells is one of the causative mechanisms of kidney fibrosis. In our study, we screened lipophilic compounds using a lipid library including approximately 200 lipids to identify those that suppressed EMT induced by a transforming growth factor (TGF)-β1 stimulus. Initial screening was performed with the immortalized HK-2 renal tubule epithelial cell line. The most promising compounds were further tested in RPTEC primary renal tubule epithelial cells. We found that the synthetic lipid AM251 suppressed two hallmark events associated with EMT, the upregulation of collagen 1A1 (*COL1A1*) and downregulation of E-cadherin. Though AM251 is known to act as an antagonist for the cannabinoid receptor type 1 (CB1) and an agonist for the G protein-coupled receptor 55 (GRP55), the suppression of EMT by AM251 was not mediated through either receptor. Microarray analyses revealed that AM251 inhibited induction of several EMT transcription factors such as *SNAIL1*, which is the key inducer of EMT, and the AP-1 transcription factors *FOSB* and *JUNB*. Activation of SMAD2/3 and p38 mitogen-activated protein kinase (MAPK) was inhibited by AM251, with greater inhibition of the latter, indicating that AM251 acted upstream of SMAD/p38 MAPK in the TGF-β signaling pathway. Our findings regarding the effects of AM251 on the TGF-β signaling pathway may inform development of a novel therapeutic agent suppressing EMT, thus preventing kidney fibrosis.

## Introduction

Progression of chronic kidney disease (CKD) can cause renal fibrosis, which impairs kidney function and can lead, in the worst-case scenario, to renal failure [1]. In renal fibrosis, myofibroblasts overproduce extracellular matrix proteins such as collagens and fibronectin [2,3]. Myofibroblasts are derived from multiple sources [1], such as by activation of interstitial fibroblasts and pericytes [4], conversion of renal tubular epithelial cells through the epithelial-mesenchymal transition (EMT) [5,6], conversion of renal endothelial cells through endothelial-mesenchymal transition (EndoMT) [7], and recruitment of circulating fibrocytes [8]. Though the importance of EMT during renal fibrosis has been debated [9,10], recent studies revealed that partial EMT, in which epithelial cells remain attached to the basement membrane but the EMT program is activated, plays an important role in promoting CKD [11,12,13]. After injury of epithelial cells, transforming growth factor (TGF)-β induces expression of two key regulators of the EMT program, the transcription factor snail family zinc finger 1 (SNAIL1/SNAI1) and twist family bHLH transcription factor 1 (TWIST1) which, in turn, cause myofibroblast proliferation by promoting secretion of growth factors including TGF-β, leading to cell cycle arrest of the epithelial cells and chronic inflammation. Therefore, inhibition of the EMT program is considered a potential mechanism for anti-fibrotic therapies.

**Fig 1:**
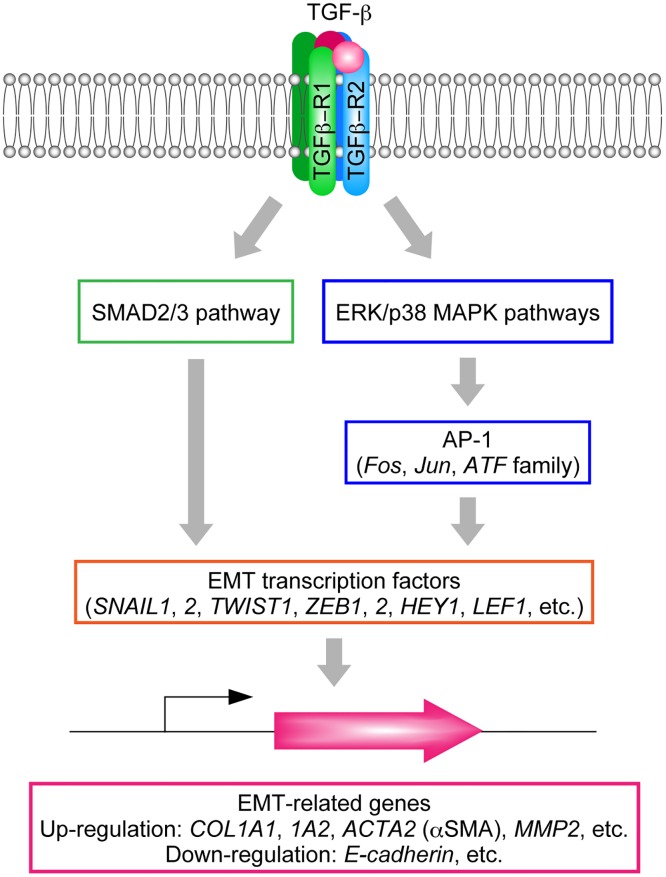
TGF-β signaling pathways and induction of EMT-related genes. Binding of TGF-β to the TGF-β receptor induces activation of SMAD2/3 and p38 MAPK via phosphorylation. The SMAD2/3 pathway directly activates EMT-related transcription factors including SNAIL1, whereas the p38 MAPK pathway activates these factors via AP-1. EMT related genes are then up- or downregulated.

TGF-β signaling has a predominant role in EMT (Fig 1). The TGF-β family includes three subtypes (TGF-β1, TGF-β2, and TGF-β3) and induces EMT through binding to TGF-β receptors (TGF-βR1–3) [14,15]. Binding of TGF-β to TGF-βR2 recruits TGF-βR1, which then activates several downstream signaling molecules or pathways, including SMAD2/3 and the following mitogen-activated protein kinases (MAPKs): extracellular regulated kinase (ERK), p38 MAPK, and Jun N-terminal kinase (JNK) [16,17,18,19,20]. These mediators in turn induce EMT transcription factors such as SNAIL1, Snail family zinc finger 2 (SNAIL2/SNAI2/SLUG), TWIST1, zinc finger E-box binding homeobox 1 (ZEB1), zinc finger E-box binding homeobox 2 (ZEB2), hes-related family bHLH transcription factor with YRPW motif 1 (HEY1), lymphoid enhancer binding factor 1 (LEF1), and transcription factor 3 (TCF3/VDIR). These events are followed by induction or suppression of EMT-related genes. Such effects include induction of genes encoding type I collagens (*COL1A1* and *COL1A2*), α smooth muscle actin (αSMA; *ACTA2*), and matrix metallopeptidase-2 (*MMP2*) and suppression of the gene encoding E-cadherin (*CDH1*) [21,22,23] (Fig 1). Expression of the key EMT inducer SNAIL1 is directly activated by SMAD2/3 [21]. Alternatively, SNAIL1 is also induced by the p38 MAPK pathway via activator protein 1 (AP-1), a heterodimeric transcription factor composed of proteins belonging to the c-Fos, c-Jun, and ATF families [24].

In this study, we screened lipophilic compounds to identify those effective at suppressing EMT in renal tubular epithelial cells *in vitro*, using a lipid library containing approximately 200 lipids. We isolated the synthetic lipid AM251 as a hit compound and further investigated the mechanisms underlying AM251-induced EMT suppression. Though AM251 is known to be an antagonist of the cannabinoid receptor type 1 (CB1) and an agonist of the G protein-coupled receptor 55 (GRP55), our results demonstrate that AM251 suppressed EMT independent of these receptors. We found that AM251 inhibited activation of SMAD2/3 and p38 MAPK and induction of *SNAIL1*. Therefore, our findings may facilitate development of future clinical applications for treating kidney fibrosis.

## Materials and Methods

### Cell Culture

The immortalized proximal tubule epithelial cell line HK-2 (American Type Culture Collection, Manassas, VA, USA) was grown in serum-free keratinocyte medium (Thermo Fisher Scientific, Waltham, MA, USA) containing 5 ng/ml recombinant human epidermal growth factor (Thermo Fisher Scientific), 0.05 mg/ml bovine pituitary extract (Thermo Fisher Scientific), 100 units/ml penicillin, and 100 μg/ml streptomycin. The primary renal proximal tubule epithelial cells RPTEC (Lonza, Basel, Switzerland) were grown in the renal epithelial growth media (REGM; Lonza) containing REGM Single Quots (Lonza). REGM Single Quots includes a mixture of supplements and growth factors (hydrocortisone, epidermal growth factor, fetal bovine serum, epinephrine, insulin, triiodothyronine, transferrin, gentamicin and amphotericin B). RPTEC cells were used at passage 3 or 4. EMT was induced by replacing the growth medium with REGM lacking REGM Single Quots but containing 2 ng/ml TGF-β1 (R&D Systems, Minneapolis, MN, USA). However, in the experiment involving TGF-β1 pretreatment, REGM containing REGM Single Quots and 2 ng/ml TGF-β1 was used to prevent cytotoxicity.

### RNA Interference

RNA interference was carried out using RNAiMAX (Thermo Fisher Scientific) by the reverse transfection method, according to the manufacturer’s instructions. The *Silencer*^®^ Select Negative Control No. 1 siRNA (Thermo Fisher Scientific) and siRNAs for the CB1 gene *CNR1* (s3260 and s3262; Thermo Fisher Scientific) were used at 20 nM.

### Screening for EMT-inhibiting Compounds

HK-2 cells were plated at 2 × 10^4^ cells per well on poly-D-lysine-coated 96-well plates overnight. TGF-β1 (2 ng/ml) and one of the compounds from SCREEN-WELL^®^ Bioactive lipid library (10 or 1 μM; 1/100 of the original concentrations in the library; Enzo Life Sciences, Farmingdale, NY, USA) were incubated with the cells for 24 h. Expression of collagen 1A1 (*COL1A1*) and, as a housekeeping gene, peptidylprolyl isomerase (*PPIA*) was then measured by real-time RT-PCR as described below.

### Real-Time Quantitative RT-PCR

To detect EMT inhibiting compounds, cells were lysed using Fastlane Cell SYBR Green Kit (Qiagen, Hilden, Germany) and subjected to real-time RT-PCR using QuantiTect SYBR Green RT-PCR Kit (Qiagen) and primers (for *COL1A1*, COL1A1-F and COL1A1-R; for *PPIA*, PPIA-F and PPIA-R; Table 1), according to the manufacturer’s instructions.

**Table 1:** Primers used in this study.

Primer	Nucleotide sequence
PPIA-F	5'-ATCTGCACTGCCAAGACTGAG-3'
PPIA-R	5'-GAAGGAATGATCTGGTGGTTAAGA-3'
COL1A1-F	5'-AAGCTTGGTCCACTTGCTTGAA-3'
COL1A1-R	5'-GAGCATTGCCTTTGATTGCTG-3'
CDH1-F	5'-GCCCATTTCCTAAAAACCTGG-3'
CDH1-R	5'-TTGGATGACACAGCGTGAGAG-3'
CNR1-F	5'-CTGAGGATGATGTACTTGCCCTGA-3'
CNR1-R	5'-CTCTTGGAGGCAGCCCTACTTG-3'
GPR55-F	5'-CCAGGAGCTGCATGGCTGTA-3'
GPR55-R	5'-GGCACACCCACAGGTCCATA-3'
SNAI1-F	5'-ACTATGCCGCGCTCTTTCCT-3'
SNAI1-R	5'-AGTCCTGTGGGGCTGATGTG-3'
SNAI2-F	5'-GCTGTAGGAACCGCCGTGTC-3'
SNAI2-R	5'-ATTTGTCATTTGGCTTCGGAGTG-3'
JUNB-F	5'-CATACACAGCTACGGGATACG-3'
JUNB-R	5'-GCTCGGTTTCAGGAGTTTG-3'
FOSB-F	5'-CTCGGCCTAGGTCACGTT-3'
FOSB-R	5'-GCCAGAGTTTCTAGAAGCAGTTT-3'
TGFR1-F	5'-AACCCTGCCTAGTGCAAGTTACAA-3'
TGFR1-R	5'-GACTAACAAATGTGCTGACCCAAAG-3'
TGFB2-F	5'-GCTGAGCCAGCCAGATATAACAAGA-3'
TGFB2-R	5'-ACAGGCAAGTAGCTGATCCCAAAC-3'
TGFB3-F	5'-GGCTACTATGCCAACTTCTGCTCA -3'
TGFB3-R	5'-AGGCAGATGCTTCAGGGTTCA-3'

In other assays, total RNA was isolated from cells using the RNeasy Plus Micro Kit (Qiagen) and converted to cDNA using the High Capacity RNA-to-cDNA Kit (Thermo Fisher Scientific). The resulting cDNAs were then subjected to real-time RT-PCR using SYBR Premix Ex Taq II (Takara, Shiga, Japan) on the 7500 Real-Time PCR System (Thermo Fisher Scientific). Primers used are listed in Table 1 (-F, forward primer; -R, reverse primer).

### Immunoblotting

After washing with phosphate-buffered saline three times, cells were lysed in cold RIPA buffer (25 mM Tris-HCl (pH 7.6), 150 mM NaCl, 1% NP-40, 1% sodium deoxycholate, and 0.1% SDS) containing Halt Protease Inhibitor Cocktail, EDTA-Free (ThermoFisher Scientific) for detection of E-cadherin and GAPDH, or Halt Protease and Phosphatase Inhibitor Cocktail (ThermoFisher Scientific) for detection of phosphorylated p38 MAPK and SMAD3 (all per manufacturer’s instructions). Samples were then treated with Lane Marker Reducing Sample Buffer (ThermoFisher Scientific; 60 mM Tris-HCl (pH 6.8), 1% SDS, 10% glycerol, and 20 mM dithiothreitol) and heated at 95°C for 10 min. Equal amounts of samples (5 μg protein per lane) were separated by SDS-PAGE and transferred to an iBlot^®^ PVDF Membrane (ThermoFisher Scientific) using the iBlot dry blotting system (ThermoFisher Scientific). The resulting membrane was incubated with ECL Prime Blocking Reagent (GE Healthcare Life Sciences, Little Chalfont, UK) for 2 h at room temperature and then with anti-E-cadherin, anti-GAPDH, anti-SMAD3, anti-phospho-SMAD3, anti-p38 MAPK, or anti-phospho-P38 MAPK antibodies (each at 1:1000 dilution; Cell Signaling, Danvers, MA, USA) for 1 h at room temperature. After washing with TBS-T (50 mM Tris-HCl (pH 7.5), 137 mM NaCl, and 0.05% Tween 20), the membrane was incubated with horseradish peroxidase-conjugated anti-rabbit IgG antibody (1:2000 dilution; GE Healthcare Life Sciences) for 2 h at room temperature, then washed three times with TBS-T. Labeling was detected using the ECL Prime Western Blotting Detection Reagent (GE Healthcare Life Sciences) on an ImageQuant LAS 4000 (GE Healthcare Life Sciences). Quantification of protein levels was performed using MultiGauge software (Fujifilm, Tokyo, Japan).

### Microarray

Total RNA was prepared using the RNeasy Plus Micro Kit (Qiagen). RNA quality was evaluated using Agilent 2100 Bioanalyzer (Agilent Technologies, Santa Clara, CA, USA). Total RNA samples with an RNA integrity number >8.0 [25] were converted to cRNAs and labeled with Cy3 using a Low Input Quick Amp Labeling Kit, One-Color (Agilent Technologies). Cy3-labeled cRNA was hybridized to a SurePrint G3 Human GE Microarray G4851B (Agilent Technologies) at 65°C for 17 h using a Gene Expression Hybridization Kit (Agilent Technologies). The microarray slide was washed, dried, and scanned with a SureScan microarray scanner G2600D (Agilent Technologies). The raw microarray image data were read and processed by Feature Extraction software 11.5.1.1 (Agilent Technologies). Data were analyzed using GeneSpring GX 12 software (Agilent Technologies). The 95^th^ percentile signal value was used to normalize microarray signals for inter-array comparisons. Genes with ≥2-fold change (increase or decrease), upon treatment of cells with TGF-β1, were subjected to hierarchical clustering analyses and enrichment pathway analyses. Hierarchical clustering analyses were performed by centroid distance method using GeneSpring GX 12.5. Enrichment pathway analyses were conducted using the MetaCore gene regulatory network database (Thomson Reuters, New York, NY, USA).

## Results

### Identification of AM251 as an EMT-inhibiting Compound

**Fig 2:**
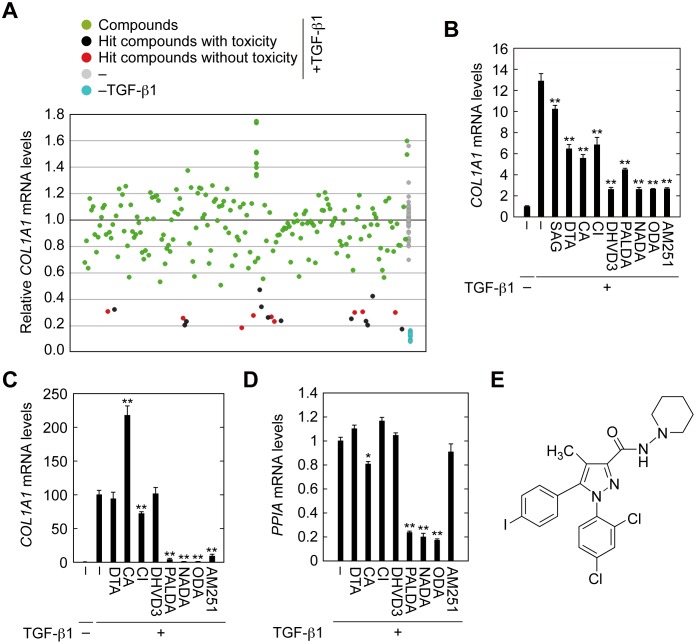
Identification of AM251 as an EMT suppressing compound. (A) HK-2 cells were incubated with 2 ng/ml TGF-β1 and individual compounds from the SCREEN-WELL^®^ Bioactive lipid library (each at a 1:100 dilution of the original library) for 24 h. Cells were lysed and subjected to real-time RT-PCR to quantitate *COL1A1* and *PPIA* mRNA levels as indicators of effects on EMT and cytotoxicity, respectively. Compounds causing ≥4-fold decreases in *PPIA* levels compared with control (no compound, +TGF-β1; gray circles) were eliminated from evaluation because of their toxicity (black circles). Compounds that decreased the ratio of expression levels of *COL1A1* to *PPIA* to ≤50% compared with control (no compound, +TGF-β1; gray circles) were selected as hit compounds (red circles). (B–D) HK-2 (B) and RPTEC (C and D) cells were incubated with 2 ng/ml TGF-β1 and individual hit compounds (1-stearoyl-2-arachidonoyl-glycerol (SAG) at 20 μM; docosatrienoic acid (DTA) at 20 μM; carbaprostacyclin (CA) at 10 μM; ciglitazone (CI) at 10 μM; 24,25-dihydroxyvitamin D_3_ (DHVD3) at 20 μM; *N*-palmitoyl dopamine (PALDA) at 10 μM; *N*-arachidonoyl dopamine (NADA) at 10 μM; *N*-oleoyl dopamine (ODA) at 10 μM; or AM251 at 10 μM) for 24 h and then subjected to real-time RT-PCR to quantitate *COL1A1* and *PPIA* mRNA levels. Values are means ± SD of the ratio of *COL1A1* to *PPIA* mRNA levels, expressed relative to the ratio in the control (no treatment) (B and C) or *PPIA* mRNA levels relative to the control (no compound + TGF-β1) (D). Data were from three independent experiments. Statistically significant differences from the control (no compound +TGF-β1) are indicated (** *P* < 0.01, Student’s *t*-test). (E) Structure of AM251.

We performed screening to identify lipophilic compounds that suppressed EMT induced by TGF-β1. For this screening, we used the renal tubular epithelial cell line HK-2 and a lipid library consisting of 196 natural and synthetic lipids. As an indicator of EMT, expression of type I collagen *COL1A1* was examined. Expression levels of *COL1A1* in cells treated with TGF-β1 were more than 10-fold higher than in those without treatment. Those compounds that reduced *COL1A1* expression to ≤50% were regarded as hit compounds (Fig 2A, red circles). We also examined expression levels of peptidylprolyl isomerase (*PPIA*) to monitor cytotoxicity of the library compounds. Compounds causing a ≥4-fold decrease in *PPIA* expression were excluded from the hit compounds (Fig 2A, black circles). We obtained nine potential hit compounds, 1-stearoyl-2-arachidonoyl-glycerol (SAG), docosatrienoic acid (DTA), carbaprostacyclin (CA), ciglitazone (CI), 24, 25-dihydroxyvitamin D_3_ (DHVD3), *N*-palmitoyl dopamine (PALDA), *N*-arachidonoyl dopamine (NADA), *N*-oleoyl dopamine (ODA), and AM251. Reproducibility testing by real-time RT-PCR confirmed that all except SAG, which caused only 20% reduction in TGF-β1-induced *COL1A1* expression, met the criteria for hit compounds (Fig 2B).

Next, the remaining eight hit compounds were subjected to secondary screening in primary renal tubular epithelial cells (RPTEC). Four compounds, DTA, CA, CI, and DHVD3, exhibited little to no EMT suppressing activity (Fig 2C). Three compounds, PALDA, NADA, and ODA, exhibited high toxicity (Fig 2D), though they also caused significant EMT suppression. Only AM251 exhibited EMT suppressing activity without toxicity and was therefore used for further analyses. The structure of AM251 is shown in Fig 2E.

**Fig 3:**
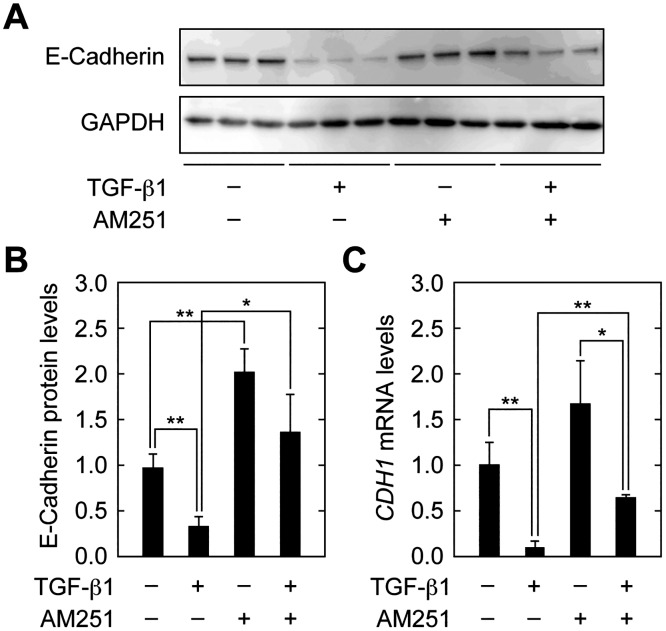
AM251 reverses TGF-β1-dependent decreases in E-cadherin expression. (A–C) RPTEC cells were incubated with 2 ng/ml TGF-β1 and/or 10 μM AM251, as indicated, for 24 h. (A) Total protein lysates were prepared and equal amounts of proteins (5 μg per sample) were separated by SDS-PAGE, followed by immunoblotting with an anti-E-cadherin antibody or an anti-GAPDH antibody as a loading control. (B) The results from (A) were quantified. Values are means ± SD of E-cadherin protein levels relative to those in cells with no treatment (TGF-β1(−) AM251(−)), from three independent experiments. (C) Total RNA was prepared and subjected to real-time RT-PCR to measure E-cadherin (*CDH1*) and *PPIA* mRNA levels. Values are means ± SD of the ratio of *CDH1* to *PPIA* mRNA levels, expressed relative to the ratio in cells with no treatment (TGF-β1(−) AM251(−)), from three independent experiments. Statistically significant differences are indicated (* *P* < 0.05, ** *P* < 0.01, Student’s *t*-test).

At the first step of EMT, cell–cell contacts between epithelial cells were disassembled. Consistent with this, downregulation of the component of adherens junctions, E-cadherin, is a well-known indicator of EMT [6,23]. TGF-β1 treatment indeed decreased E-cadherin protein levels to 34.2% of those in control cells (Fig 3A and 3B). AM251 caused an increase in E-cadherin levels both in the presence and absence of TGF-β1. Similar results were obtained for mRNA levels of the E-cadherin gene (*CDH1*) examined by real-time RT-PCR (Fig 3C). Thus, AM251 reversed the downregulation of E-cadherin caused by TGF-β1.

**Fig 4:**
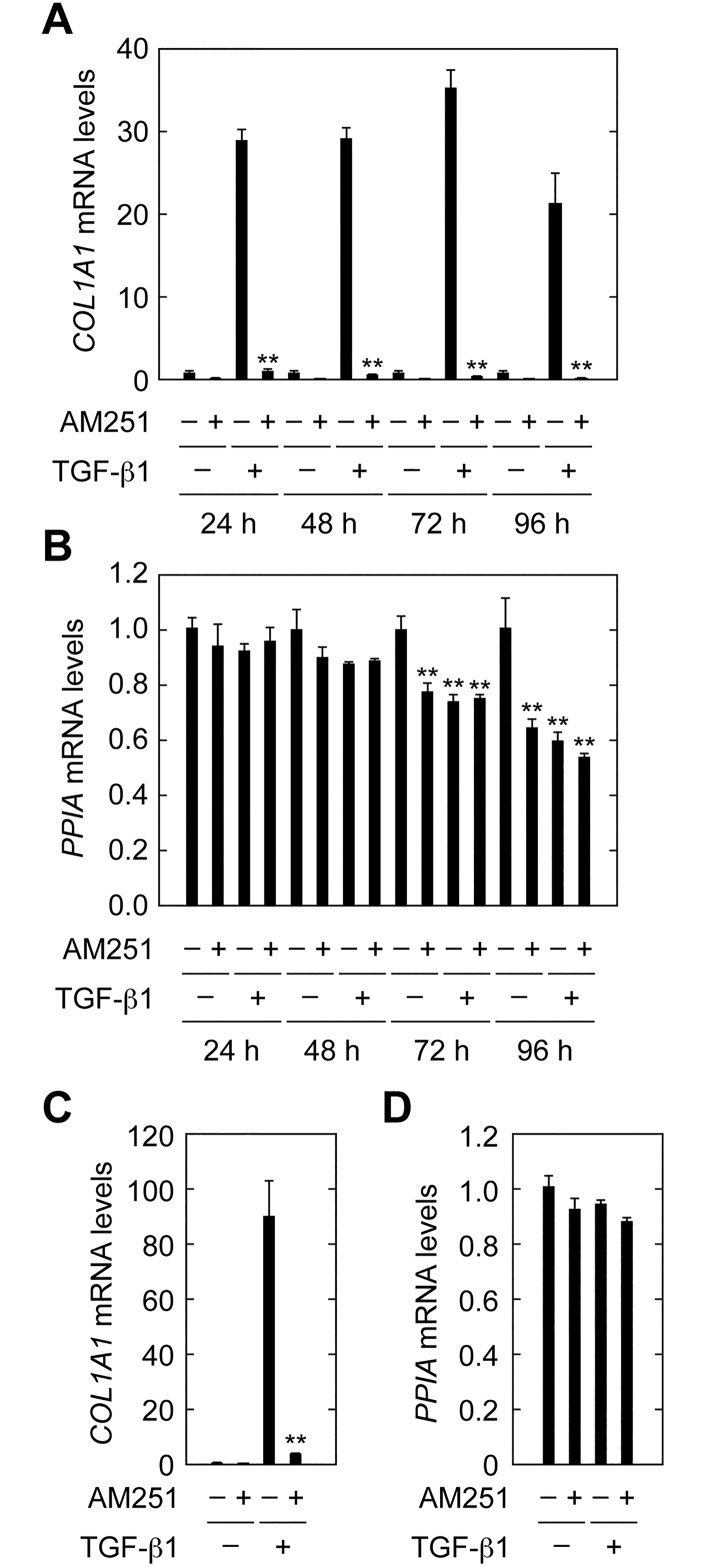
AM251 suppresses *COL1A1* expression pre-induced by TGF-β1. (A and B) RPTEC cells were cultured in REGM medium containing 2 ng/ml TGF-β1 and/or 10 μM AM251, as indicated, for 24, 48, 72, or 96 h. Total RNA was prepared and subjected to real-time RT-PCR to measure *COL1A1* and *PPIA* mRNAs. Values are means ± SD of the ratio of *COL1A1* to *PPIA* mRNA levels, expressed relative to the ratio in the control (no treatment) (A) or *PPIA* mRNA levels relative to the control (no treatment) (B), from three independent experiments. Statistically significant differences from the control (A, without AM251; B, no treatment) are indicated (** *P* < 0.01, Student’s *t*-test). (C and D) RPTEC cells were incubated with 2 ng/ml TGF-β1 for 96 h in REGM medium containing REGM Single Quots and incubated with 2 ng/ml TGF-β1 and/or 10 μM AM251, as indicated, for the following 24 h in REGM medium. Total RNA was prepared and subjected to real-time RT-PCR to measure *COL1A1* and *PPIA*. Relative mRNA levels of *COL1A1* (C) and *PPIA* (D) were determined as described for (A) and (B), respectively.

In the experiments described above, cells were treated with TGF-β1 and AM251 for 24 h and then subjected to gene expression analyses. We also examined effects of longer AM251 treatment times (48, 72 and 96 h) on TGF-β1-induced *COL1A1* expression. Cell toxicity was also evaluated by measuring expression of the housekeeping gene *PPIA*. AM251 strongly suppressed *COL1A1* induction at all treatment times, although there was some cytotoxicity with TGF-β1 and AM251 with the 72 and 96 h treatment conditions (Fig 4A and 4B). We next examined whether AM251 could suppress *COL1A1* induction caused by TGF-β1 pretreatment for 96 h. Treatment with AM251 for 24 h following TGF-β1 pretreatment strongly suppressed *COL1A1* expression (Fig 4C and 4D). These results indicate that the suppression by AM251 was sustained for long periods and was effective even when *COL1A1* expression had already been induced.

### AM251 Suppresses EMT Independent of CB1 or GRP55

**Fig 5:**
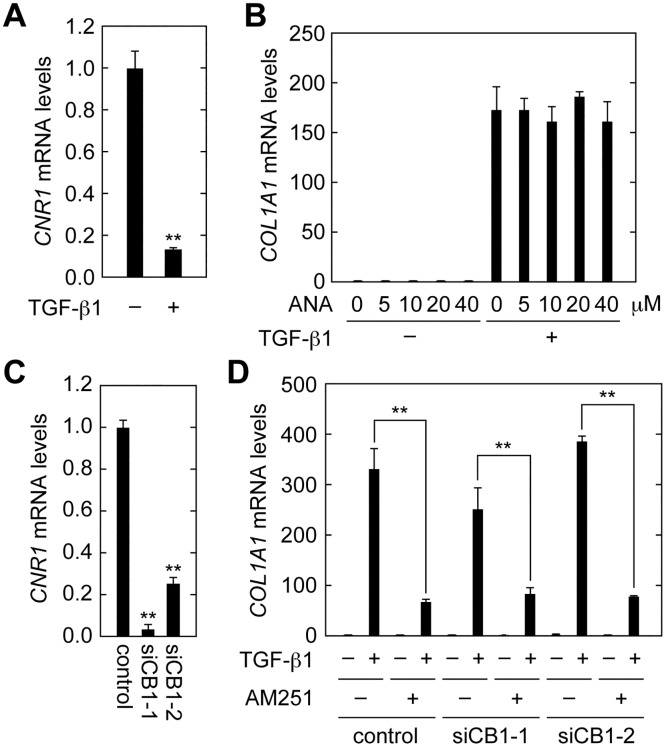
CB1 is not involved in EMT suppression in RPTEC cells. (A) RPTEC cells were incubated with 2 ng/ml TGF-β1 for 24 h. Total RNA was prepared and subjected to real-time RT-PCR to measure CB1 (*CNR1*) and *PPIA* mRNA levels. Values are means ± SD of the ratio of *CNR1* to *PPIA* mRNA levels, expressed relative to the ratio in the control, from three independent experiments. (B) RPTEC cells were incubated with 2 ng/ml TGF-β1 and the CB1 agonist anandamide (ANA) at the indicated concentrations for 24 h. Relative mRNA levels of *COL1A1* were determined as for (A). (C) RPTEC cells were treated with control siRNA or a selective siRNA for *CNR1* (siCB1-1 or siCB1-2) for 72 h. Relative mRNA levels of *CNR1* were determined as for (A). (D) RPTEC cells were treated with control siRNA or selective siRNA for *CNR1* (siCB1-1 or siCB1-2) for 48 h and then incubated with 2 ng/ml TGF-β1 and 10 μM AM251 for another 24 h. Relative mRNA levels of *COL1A1* were determined as for (A). Statistically significant differences are indicated (** *P* < 0.01, Student’s *t*-test).

AM251 is known as an antagonist of cannabinoid receptor type 1 (CB1) or an agonist of the G protein-coupled receptor 55 (GPR55) [26,27]. Both CB1 and GPR55 are known as cannabinoid receptors [27,28,29] and are activated by exogenous plant cannabinoids, as well as by the endocannabinoids 2-arachidonoylglycerol and anandamide [27,28,29]. GPR55 is also activated by lysophosphatidylinositol [30]. To examine whether the effects of AM251 on EMT suppression were mediated through these receptors, we first measured mRNA levels of the CB1 gene (*CNR1*) in RPTEC cells. Real-time RT-PCR revealed that *CNR1* mRNA levels were decreased in cells treated with TGF-β1 (Fig 5A). When cells were treated with anandamide (ANA), the agonist of CB1 and GPR55, expression of *COL1A1* mRNA was unchanged in either the presence or absence of TGF-β1 (Fig 5B). We next performed CB1 knockdown analyses using two independent siRNAs specific for the CB1 gene *CNR1* (siCB1-1 and siCB1-2). Both siRNAs significantly reduced *CNR1* mRNAs levels (to 3% by siCB1-1 and to 25% by siCB1-2) (Fig 5C). However, these siRNAs had no effect on TGF-β1-induced *COL1A1* expression, either in the presence or absence of AM251 (Fig 5D).

**Fig 6:**
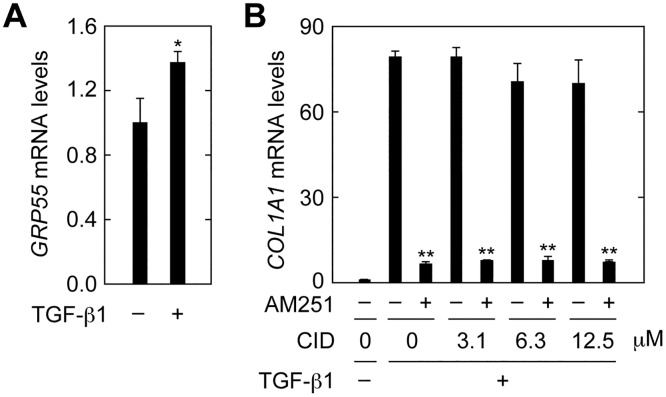
GPR55 is not involved in EMT suppression in RPTEC cells. (A) RPTEC cells were incubated with 2 ng/ml TGF-β1 for 24 h. Total RNA was prepared and subjected to real-time RT-PCR to measure *GRP55* and *PPIA* mRNA levels. Values are means ± SD of the ratio of *GRP55* to *PPIA* mRNA levels, expressed relative to the ratio in the control (no treatment), from three independent experiments. (B) RPTEC cells were incubated with 2 ng/ml TGF-β1 in the presence or absence of the GRP55 antagonist CID16020046 (CID; Tocris Bioscience, Minneapolis, MN, USA) at the indicated concentrations and 10 μM AM251 for 24 h. Relative mRNA levels of *COL1A1* were determined as for (A). Statistically significant differences are indicated (* *P* < 0.05, ** *P* < 0.01, Student’s *t*-test).

Expression of *GRP55* mRNA was slightly increased in cells treated with TGF-β1 (Fig 6A). When cells were treated with the selective GRP55 antagonist CID16020046 (CID) [31] at increasing concentrations, the expression of *COL1A1* induced by TGF-β1 was not affected at any concentration, with or without AM251 (Fig 6B). From these results, we conclude that the EMT suppressing activity of AM251 was not mediated through CB1 or GRP55.

### AM251 Suppresses Induction of *SNAIL1*, *FOSB*, and *JUNB* Expression

**Fig 7:**
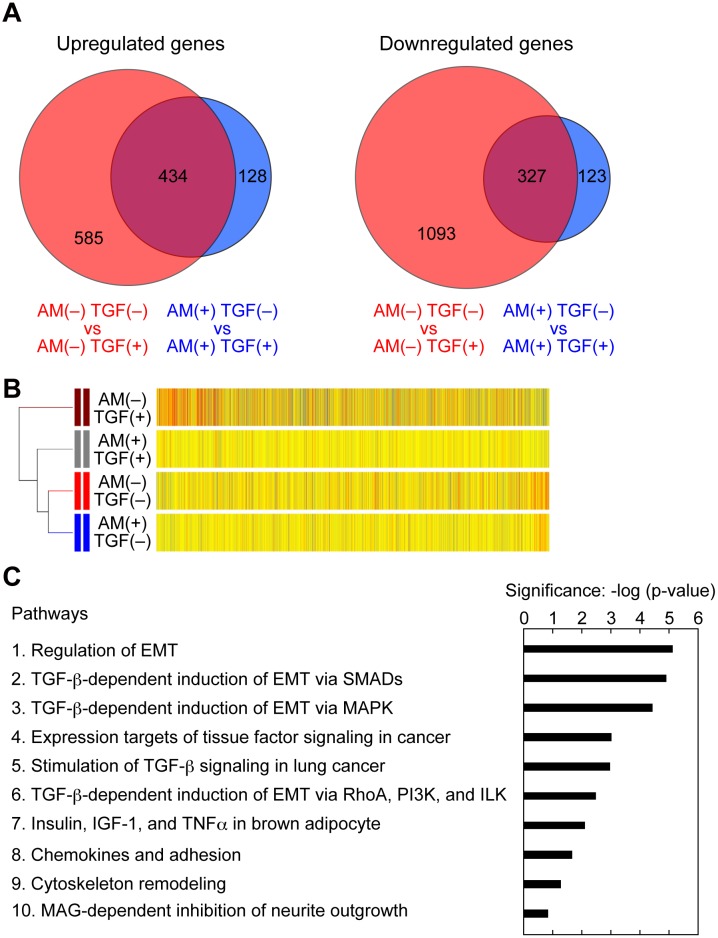
AM251 inhibits EMT with specificity. (A–C) RPTEC cells were incubated with or without 2 ng/ml TGF-β1 (TGF) in the presence or absence of 10 μM AM251 (AM) for 24 h. Total RNA was isolated from three independent samples, pooled, and subjected to microarray analyses. (A) Comparison of the microarray data was conducted on the data from AM251(−) TGF-β1(−) versus those from AM251(−) TGF-β1(+) conditions (red circles); and from AM251(+) TGF-β1(−) versus those from AM251(+) TGF-β1(+) conditions (blue circles). Numbers of upregulated (≥2-fold; left panels) and downregulated (≥2-fold; right panels) genes are depicted as Venn diagrams. (B) Hierarchical clustering analyses were performed using genes upregulated by ≥2-fold upon treatment with TGF-β1 by the centroid distance method. Yellow bars indicate that the values are similar to the averages of four different conditions. Red and blue bars indicate that the values are higher and lower than the averages, respectively, with the color strengths representing the degrees of the values. (C) Enrichment pathway analyses were conducted using genes with ≥2-fold change upon treatment with TGF-β1 and the MetaCore gene regulatory network database. The top 10 pathways are shown. PI3K, phosphoinositide 3-kinase; ILK, integrin-linked kinase; IGF, insulin-like growth factor; TNFα, tumor necrosis factor α; MAG, myelin-associated glycoprotein.

To elucidate the molecular mechanisms underlying AM251-induced EMT suppression, we next performed comprehensive gene expression analyses by microarray. For this purpose, we prepared total RNA samples from cells treated under 4 different conditions: AM251(−) TGF-β1(−), AM251(−) TGF-β1(+), AM251(+) TGF-β1(−), and AM251(+) TGF-β1(+). We first compared the results of AM251(−) TGF-β1(−) and AM251(−) TGF-β1(+). TGF-β1 treatment caused upregulation (≥2-fold increase) of 1,019 genes and downregulation (≥2-fold decrease) of 1,420 genes (Fig 7A, red circles). In contrast, in the presence of AM251, TGF-β1 treatment resulted in upregulation of 562 genes and downregulation of 450 genes (Fig 7A, blue circles). This indicates that AM251 suppressed upregulation of 585 of the 1,019 genes and downregulation of 1,093 genes among the 1,420 that had been induced by TGF-β1 alone. Thus, AM251 suppressed 68.8% of the gene expression changes caused by TGF-β1.

To compare gene expression patterns among cells subjected to different conditions, we performed hierarchical clustering analyses using genes increased by ≥2-fold upon treatment with TGF-β1 (Fig 7B). The gene expression pattern under untreated conditions (AM251(−) TGF-β1(−)) was most similar to that with only AM251 treatment (AM251(+) TGF-β1(−)). This result suggests that AM251 treatment did not globally affect gene expression. The gene expression pattern under TGF-β1 treatment conditions (AM251(−) TGF-β1(+)) differed the most from that under the untreated conditions, indicating that many genes observed were under control of TGF-β1 signaling. Co-treatment of cells with AM251 and TGF-β1 caused suppression of many of the TGF-β1-induced gene expression changes, that is, the gene expression pattern of AM251(+) TGF-β1(+) was more similar to that of AM251(−) TGF-β1(−) than AM251(−) TGF-β1(+).

We next performed pathway analyses using the MetaCore gene regulatory network database, in which approximately 1,500 pathways are registered, to reveal which pathways were affected by AM251. We analyzed genes with ≥2-fold expression changes under AM251(+) TGF-β1(+) conditions, compared with those under AM251(−) TGF-β1(+) conditions. The list of the 10 pathways most affected by AM251 treatment in the presence of TGF-β1 included 5 EMT- or TGF-β-related pathways (Fig 7C). Others in this list included a pathway involved in adhesion and that involved in cytoskeleton remodeling. Because changes in adhesion and the cytoskeleton are induced by EMT, these pathways can also be considered to be EMT-related. These results suggest that AM251 did not affect pathways globally, but instead affected EMT/TGF-β-related pathways relatively specifically.

**Fig 8:**
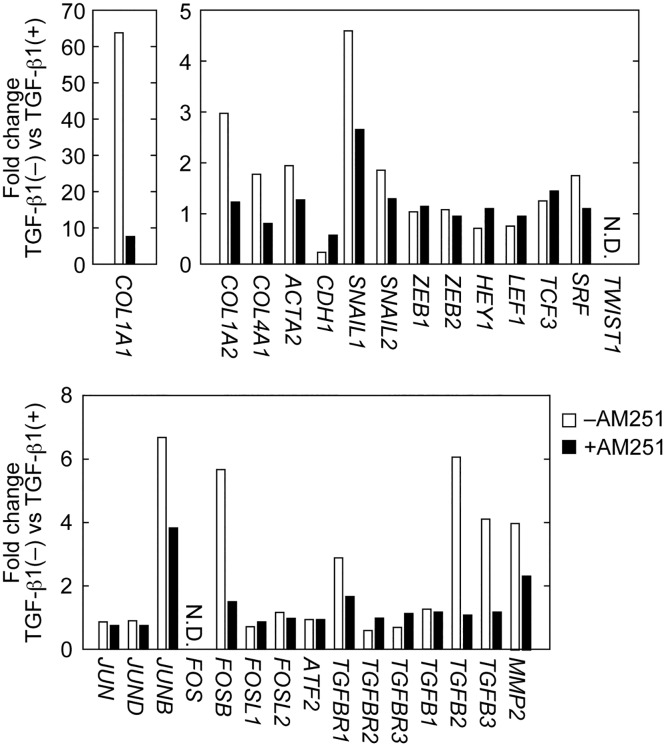
AM251 reverses TGF-β1-induced changes in expression of EMT-related genes. RPTEC cells were incubated with or without 2 ng/ml TGF-β1 in the presence or absence of 10 μM AM251 for 24 h. Total RNA was isolated from three independent samples, pooled, and subjected to microarray analyses. Values represent gene expression changes upon treatment with TGF-β1. N.D., not detected.

Among the microarray data, some EMT-related genes that were identified are shown in Fig 8. These included type I and type IV collagens (*COL1A1*, *COL1A2*, and *COL4A1*), αSMA (*ACTA2*), E-cadherin (*CDH1*), transcription factors (*SNAIL1*, *SNAIL2*, *ZEB1*, *ZEB2*, *HEY1*, *LEF1*, *TCF3*, *SRF*, and *TWIST1*), AP-1 transcription factors (*JUN*, *JUND*, *JUNB*, *FOS*, *JUNB*, *FOSL1*, *FOSL2*, and *ATF2*), TGF-β receptors (*TGFBR1*, *TGFBR2*, and *TGFBR3*), TGF-β family members (*TGFB1*, *TGFB2*, and *TGFB3*), and the matrix metallopeptidase *MMP2*. Consistent with the results of real-time RT-PCR (Figs 2C and 3C), TGF-β1 treatment caused an increase in *COL1A1* and a decrease in E-cadherin (*CDH1*) expression; AM251 reversed these effects (Fig 8). As with *COL1A1*, expression of *COL1A2*, *COL4A1*, *ACTA2* (αSMA), *SNAIL1*, *JUNB*, *TGFBR1*, *TGFB2*, *TGFB3*, and *MMP2* were increased by TGF-β1 treatment and suppressed by AM251 (Fig 8).

**Fig 9:**
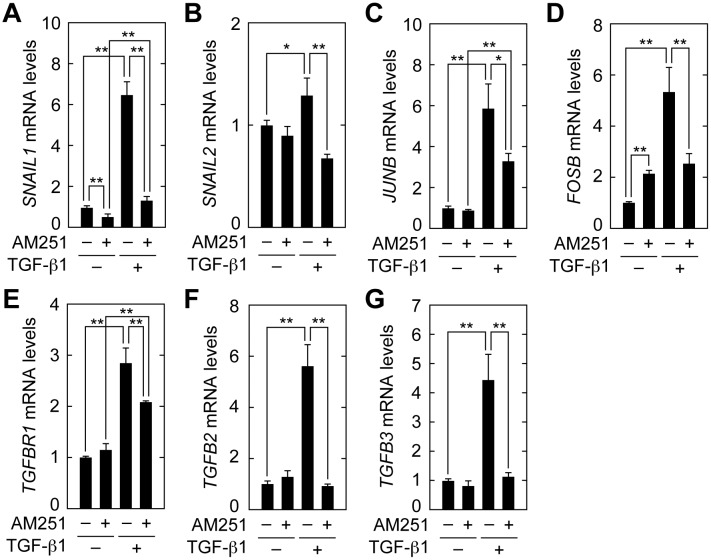
AM251 suppresses induction of *SNAILs*, *JUNB*, *FOSB*, *TGFR1*, and *TGFBs*. RPTEC cells were incubated with or without 2 ng/ml TGF-β1 in the presence or absence of 10 μM AM251 for 24 h. Total RNA was prepared and subjected to real-time RT-PCR to measure *PPIA* and *SNAIL1* (A), *SNAIL2* (B), *JUNB* (C), *FOSB* (D), *TGFBR1* (E), *TGFB2* (F), and *TGFB3* (G) mRNA levels. Values are means ± SD of the ratio of each gene to *PPIA* mRNA levels, expressed relative to the ratio in the control (no treatment), from three independent experiments. Statistically significant differences are indicated (* *P* < 0.05, ** *P* < 0.01, Student’s *t*-test).

We next confirmed the results of the microarray analyses by real-time RT-PCR. Expression of the key transcription factor involved in EMT, *SNAIL1*, was increased 6.5-fold in cells treated with TGF-β1, whereas AM251 suppressed its expression to nearly basal levels (Fig 9A). *SNAIL2* expression was also induced by TGF-β1, albeit to a lesser extent than *SNAIL1* (Fig 9B). AM251, similarly, reversed *SNAIL2* expression to basal levels (Fig 9B). The AP-1 components (*JUNB* and *FOSB*), transcription factors upstream from *SNAIL1*, were increased by 5–6 fold in cells treated with TGF-β1. AM251 treatment decreased their expression to about 50% (Fig 9C and 9D). The expression level of the TGF-β receptor *TGFBR1* was increased 2.9-fold by TGF-β1 treatment and reduced to 79% by AM251 (Fig 9E). TGF-β1 treatment caused a large increase in expression of TGF-β family members *TGFB2* (5.7-fold) and *TGFB3* (4.4-fold); AM251 almost completely reversed these effects (Fig 8F and 8G). Thus, the results of microarray analyses are consistent with those of real-time RT-PCR.

### AM251 Inhibits Activation of the SMAD2/3 and p38 MAPK Pathways

**Fig 10:**
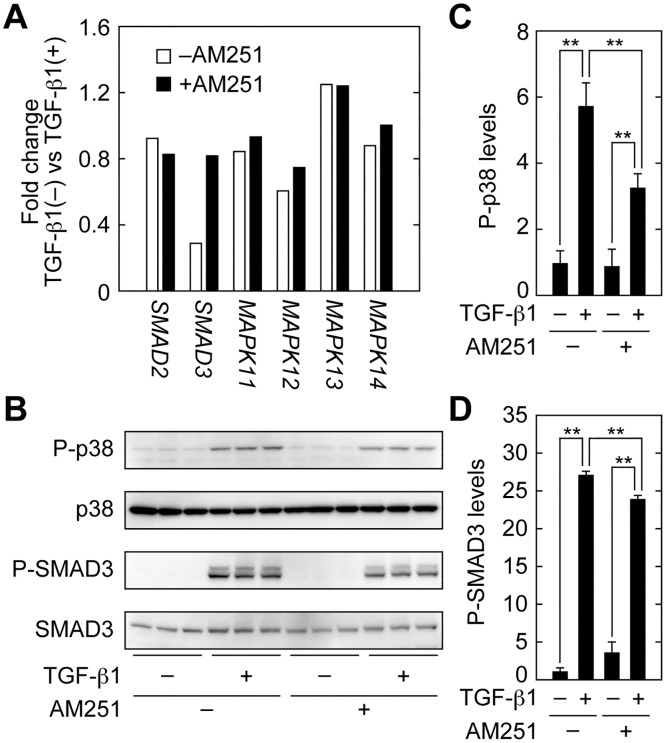
AM251 inhibits the SMAD2/3 and p38 MAPK signaling pathways. (A) RPTEC cells were incubated with or without 2 ng/ml TGF-β1 in the presence or absence of 10 μM AM251 for 24 h. (A) Total RNA was isolated from three independent samples, pooled, and subjected to microarray analyses. Values for *SMAD2*, *SMAD3*, *MAPK11* (p38β), *MAPK12* (p38γ), *MAPK13* (p38δ), and *MAPK14* (p38) represent their gene expression changes in cells treated with TGF-β1. (B–D) RPTEC cells were incubated with or without 2 ng/ml TGF-β1 in the presence or absence of 10 μM AM251 for 1 h. (B) Total protein lysates were prepared, and equal amounts of protein (5 μg per sample) were separated by SDS-PAGE, followed by immunoblotting with anti-phopho-p38 (P-p38), anti-p38, anti-phospho-SMAD3 (P-SMAD3), or anti-SMAD3 antibodies. (C and D) The results from (B) were quantified. Values are means ± SD of phopho-p38 (C) or phospho-SMAD3 (D) levels relative to those in cells with no treatment (TGF-β1(−) AM251(−)), from three independent experiments. Statistically significant differences are indicated (** *P* < 0.01, Student’s *t*-test).

To determine the site of action of AM251, we next focused on the most upstream components of TGF-β signaling. TGF-β signaling begins with activation of SMAD2/3 and MAPKs by phosphorylation (Fig 1) [16,17,18,19,20]. Microarray analyses indicated that AM251 did not cause downregulation of *SMAD2*, *SMAD3*, *MAPK11* (p38β), *MAPK12* (p38γ), *MAPK13* (p38δ), or *MAPK14* (p38) (Fig 10A). Because SMAD2/3 and MAPKs are often regulated at the level of activity rather than at the level of expression, we next examined the activation status of SMAD3 and p38 MAPK by detecting their phosphorylated (active) forms. TGF-β1 treatment caused a 5.7-fold increase in phospho-p38 MAPK levels (Fig 10B and 10C). Simultaneous treatment of cells with AM251 decreased phospho-p38 MAPK levels to nearly half of this stimulated value. SMAD3 phosphorylation was increased by 27-fold in cells treated with TGF-β1 (Fig 10B and 10D). AM251 reduced phospho-SMAD3 levels, but only slightly (an approximately 10% decrease). Therefore, AM251 inhibited activation of both SMAD2/3 and p38 MAPK pathways, though its effect on p38 MAPK was greater.

## Discussion

In our study, we screened lipid compounds to identify those that suppressed EMT induced by a TGF-β1 stimulus in renal tubular epithelial cells. We identified AM251, a known CB1 antagonist and GPR55 agonist, as an EMT suppressor. To our knowledge, our study is the first to report that AM251 affects EMT in renal tubular epithelial cells. However, protection against hyperglycemia-induced glomerular hypertrophy and fibrosis by AM251 was shown in a streptozotocin-induced diabetic rat model [32]. In that study, administration of AM251 caused decreased fibronectin, TGF-β1, TNFα, and IL-6 levels, as well as PPARγ activation. However, in another report, administration of AM251 in an obese rat model did not affect kidney fibrosis, although it improved albuminuria and renal tubular structure [33]. Thus, the effects of AM251 seem to depend on the fibrosis model. It is possible that the differences among models with respect to myofibroblast conversion, such as in EMT, EndoMT, or other processes, are responsible for this discrepancy. In our study, however, we found that AM251 was able to suppress fibrosis via EMT.

Regarding the relationship between CB1 and renal fibrosis, it was reported that, in a renal fibrosis model induced by unilateral ureteral obstruction (UUO), the fibrotic area was reduced to about 2/3 that in wild-type mice by CB1 (*CNR1*) gene knockout [34]. In this renal fibrosis model, CB1 and its ligand 2-arachidonoylglycerol were increased, suggesting that CB1 signaling was stimulated. CB1 expression was increased in myofibroblasts upon TGF-β1 treatment, suggesting that CB1 was involved in myofibroblast activation [34]. Furthermore, in the streptozotocin-induced diabetic rat model, the effect of AM251 on kidney fibrosis was suggested as being mediated by CB1, because antisense RNA against CB1 (*CNR1*) had effects similar to those of AM251 treatment [32]. However, the involvement of CB1 in EMT had not been previously examined. In our study, we demonstrated that neither treatment with anandamide nor knockdown of *CB1* affected *COL1A1* expression in renal proximal tubule epithelial cells (Fig 5B and 5D). Furthermore, *CB1* itself was downregulated by TGF-β1 treatment (Fig 5A). These results indicate that CB1 was not involved in EMT. It is possible that CB1 is involved in renal fibrosis at step(s) other than EMT.

AM251 acted upstream of SMAD2/3 and p38 MAPK (Fig 10), although its exact site of action is unclear. AM251 had a stronger effect on p38 MAPK than on SMAD2/3. Therefore, we speculated that inhibition of the p38 MAPK pathway by AM251 contributed more to its EMT suppression than did inhibition of the SMAD2/3 pathway. Downstream of p38 MAPK, expression of the AP-1 components *JUNB* and *FOSB* was decreased by AM251 treatment (Figs 8 and 9). In addition, expression of the key transcription factor for EMT, *SNAIL1*, was also decreased (Figs 8 and 9). In *SNAIL1* knockout mice, renal fibroses (using the UUO model) was decreased [11]. The decrease in *SNAIL1* may have caused downregulation of many of the EMT genes observed here (Figs 8 and 9).

There are currently no efficient drugs to prevent tubular fibrosis and EMT. AM251 suppressed pre-induced *COL1A1* expression (Fig 4C). Therefore, AM251 may have potential value in both prevention and mitigation of fibrosis. In addition to the kidney, fibrosis can occur in other tissues such as liver and lung, and EMT is involved not only in fibrosis but also in cancer progression [22,23]. In a liver fibrosis model, SNAIL1 was activated and liver fibrosis was suppressed in *SNAIL1* knockout mice [35]. Thus, it is possible that inhibition of SNAIL1 can reduce not only kidney fibrosis but also that occurring elsewhere. Our findings may inform development of future drugs for treating fibrosis in the kidney and other tissues, and AM251 is a suitable lead compound for these efforts.
